# Sulphide donors affect the expression of mucin and sulphide detoxification genes in the mucosal organs of Atlantic salmon (*Salmo salar*)

**DOI:** 10.3389/fphys.2022.1083672

**Published:** 2022-12-13

**Authors:** Hanna Ross D. Alipio, Nora Albaladejo-Riad, Carlo C. Lazado

**Affiliations:** ^1^ Nofima, The Norwegian Institute of Food, Fisheries and Aquaculture Research, Ås, Norway; ^2^ Aquaculture and Fisheries Group, Wageningen University and Research, Wageningen, Netherlands; ^3^ Immunobiology for Aquaculture Group, Department of Cell Biology and Histology, Faculty of Biology, University of Murcia, Murcia, Spain

**Keywords:** aquaculture, fish, hydrogen sulphide, immunotoxicology, mucosal immunity, mucus, mucins, recirculating aquaculture system

## Abstract

Hydrogen sulphide (H_2_S) is a gas that affects mucosal functions in mammals. However, its detrimental effects are less understood in fish despite being known to cause mass mortality. Here we used explant models to demonstrate the transcriptional responses of Atlantic salmon (*Salmo salar*) mucosa to the sulphide donor sodium hydrosulphide (NaHS). The study focused on two groups of genes: those encoding for sulphide detoxification and those for mucins. Moreover, we performed pharmacological studies by exposing the organ explants to mucus-interfering compounds and consequently exposed them to a sulphide donor. Exposure to NaHS significantly affected the expression of *sulphide:quinone oxidoreductase* (*sqor1*, *sqor2*) and mucin-encoding genes (*muc5ac*, *muc5b*). The general profile indicated that NaHS upregulated the expression of sulphide detoxification genes while a significant downregulation was observed with mucins. These expression profiles were seen in both organ explant models. Pharmacological stimulation and inhibition of mucus production used acetylcholine (ACh) and niflumic acid (NFA), respectively. This led to a significant regulation of the two groups of marker genes in the gills and olfactory rosette explants. Treatment of the mucosal organ explants with the mucus-interfering compounds showed that low dose NFA triggered more substantial changes while a dose-dependent response could not be established with ACh. Pharmacological interference demonstrated that mucins played a crucial role in mucosal protection against H_2_S toxicity. These results offer insights into how a sulphide donor interfered with mucosal responses of Atlantic salmon and are expected to contribute to our understanding of the least explored H_2_S-fish interactions—particularly at the mucosa.

## 1 Introduction

Fish are exposed to several chemical stressors and toxicants during their lifetime, which may cause damage and adverse effects to their physiological functions. One of these environmental toxicants is hydrogen sulphide (H_2_S)—a naturally occurring compound characterised by the odour of rotten egg (Harbison et al., 2015). The gas is mainly produced by sulphate-reducing bacteria (SRB) using sulphate (SO_4_^2-^) as a terminal electron acceptor in the degradation of organic matter. This directly reduces it to bisulphide (HS^−^) to produce H_2_S under anaerobic conditions (Kiemer et al., 1995; Muyzer and Stams, 2008; Abdollahi and Hosseini, 2014; Dalsgaard, 2019).

Exposure to high levels of H_2_S can affect cellular energy production and respiration both in humans and fish (Kiemer et al., 1995; Muyzer and Stams, 2008; Abdollahi and Hosseini, 2014; Dalsgaard, 2019). H_2_S-related mortality has become a major issue in Atlantic salmon (*Salmo salar*) farming in recent years especially in land-based production that use water recirculation technology. This risk is high in saline systems because marine water contains more sulphates, which have been implicated in H_2_S production (Sommerset et al., 2020). Recent evidence suggests that H_2_S production can also occur in freshwater systems though the risk is not as high as in a saline environment (Rojas-Tirado et al., 2021).

There is a limited knowledge on how H_2_S affects salmon physiology. A previous study found that acute and chronic exposure to sub-lethal levels of H_2_S in salmon resulted in gill damage characterised by clubbing and thickening of the lamella; there was also liver damage including hepatic necrosis and diffuse vascular degeneration (Kiemer et al., 1995). High levels of H_2_S may affect the fish health and robustness and can result to mass mortalities in the worst case (Hjeltnes et al., 2017; Dalsgaard, 2019; Sommerset et al., 2020). Many research studies have been done to explain its formation in closed systems (Letelier-Gordo et al., 2020; Rojas-Tirado et al., 2021; Bergstedt et al., 2022), however, physiological and immunological studies designed to understand the mechanism of H_2_S in fish are rare.

The H_2_S challenge is particularly relevant for mucosal organs because they are exposed constantly to the aquatic environment. Fish mucosal organs such as the gills, gut, skin and olfactory organs are considered the first line of defence against external threats and have a direct role in fish immunological function (Verburg-Van Kemenade et al., 2009; Lazado and Caipang, 2014; Castro and Tafalla, 2015). Mucosal surfaces are lined with a mucus layer, i.e., a viscous fluid composed of mucins, lysozymes, immunoglobulins, complex mixture of proteins, ions, lipids, and other components (Gomez et al., 2013). It is mainly produced and secreted by the mucus cells (goblet cells) that occupy the surface of the epithelium (Verburg-Van Kemenade et al., 2009; Vejlsted, 2010; Castro and Tafalla, 2015; Cabillon and Lazado, 2019). In mammalian studies, H_2_S targets the mucosal organs especially the olfactory organs (Hirsch and Zavala, 1999). In rodents, highly localised nasal respiratory lesions characterised by infiltration with inflammatory cells were seen after a single three-hour exposure to 200 ppm H_2_S (Roberts et al., 2008). Sub-lethal concentration of sulphide donors (1 mM sodium hydrosulphide, NaHS) upregulated the expression of mucin genes (muc5 and muc1) in A549 human pulmonary epithelial cells and caused mucus hypersecretion (Unuma et al., 2019). H_2_S exposure induced tracheal inflammatory injury, apoptosis, pyroptosis, and necroptosis that eventually led to excessive mucus secretions and oxidative stress in chicken (Chen et al., 2019; Li et al., 2020; Song et al., 2021).

In Atlantic salmon, a recent study reported the first ever transcriptome-wide response of nasal leukocytes to two forms of sulphide donors: the salt sodium hydrosulfide (NaHS) and the organic analogue morpholin-4-ium 4-methoxyphenyl (morpholino) phosphinodithioate (GYY4137) (Cabillon and Lazado, 2022). NaHS is a fast-release sulphide donor that instantly transforms to H_2_S upon exposure to aqueous solution. GYY4137 is a slow-releasing sulphide donor, which takes at least an hour to be transformed into H_2_S (Li et al., 2008). The previous study found that exposure to these exogenous H_2_S donors is less likely to affect the cell viability of Atlantic salmon nasal leukocytes but can influence oxidative stress, innate and adaptive immunity, and interleukin signalling—particularly at higher concentrations (Cabillon and Lazado, 2022). Although abundant research has been done to elucidate the mechanisms of H_2_S in mammals and other vertebrate animals, studies in fish mucosa-H_2_S interactions are quite limited.

This study explored the molecular responses of Atlantic salmon mucosa to H_2_S. We utilised mucosal organ explants from the gills and olfactory organ of Atlantic salmon to study the mucosal consequences of H_2_S. These two mucosal organs were selected as models based on previous studies in salmon showing their responsiveness to H_2_S (Kiemer et al., 1995; Cabillon and Lazado, 2022). We focused on how H_2_S, in the form of a sulphide donor NaHS, affected the transcription of important genes encoding sulphide detoxification and mucins. This study further explored the role of mucus in H_2_S-mucosa interactions, and this was achieved by pharmacological treatment of the explants either to stimulate or inhibit mucus production prior to NaHS exposure.

## 2 Materials & methods

### 2.1 Ethics statement

The experiment was conducted as part of the routine fish production at the Norwegian Institute for Water Research (Solbergstrand, Norway). The maintenance of stock animals for experiments was in accordance with the Guidelines of the EU-legislation (2010/63/EU) as well as the Norwegian legislation on animal experimentation and was approved by the Norwegian Animal Research Authority. The experimental fish from the production stock used in the study were not subjected to any pain or distress, and they were killed solely for the use of their tissues in this experiment. Thus, approval from the Norwegian Food Safety Authority was not required.

### 2.2 Experimental fish

Atlantic salmon (*Salmo salar*) smolts, weighing approximately 100–150 g, were sourced from Norwegian Institute for Water Research (NIVA) in Solbergstrand. At the station, the smolts were reared in a seawater flow through system under the following husbandry conditions: salinity at 35 ppt, temperature at 12.8 ± 0.6°C, pH at 7.9 ± 1, dissolved oxygen >90% saturation and photoperiod at 24 h light: dark cycle. The fish were fed a commercial diet twice per day and deprived of food 24 h prior to collection of the explants.

### 2.3 Mucosal organ explants

Four salmon were humanely euthanised using an overdose of AQUI-S^®^ (MSD Animal Health, Drammen, Norway) for 5 minutes, and the weight was recorded. The gills were exposed by removing the operculum, and the entire second gill arch from both sides was excised and immediately placed in a sterile 6-well plate with chilled wash medium containing Leibovitz’s L-15 medium with 5% foetal bovine serum (FBS), 1% 100× antibiotic antimycotic solution (AAS), 1% 1 M 4-(2-hydroxyethyl)-1-piperazineethanesulfonic acid buffer (HEPES), and 0.2% 5000 IU/ml heparin (Lazado and Voldvik, 2020). The gills were perfused with sterile 1× phosphate buffered saline (PBS) through the gill arch until they blanched, thus indicating the elimination of most blood (Lazado and Voldvik, 2020). The gills were further washed with the wash medium and cut into smaller fragments approximately 1–2 mm in size. The nostrils were carefully removed to expose the olfactory rosettes. The rosettes from both sides were collected and placed in the same chilled wash media as in the gills. Each rosette was cut bilaterally, thus giving four olfactory rosette fragments from each individual fish. Six extra fish were used for the collection of olfactory rosettes to adequately carry out the treatments. Both tissue fragments were in the wash media before they were transplanted onto the culture plates.

To facilitate adhesion, the tissue explant was placed in the centre of each well of the 12-well CellBIND™ (Corning, United States); 50 µL of FBS was added to sufficiently cover the tissue fragment. This was then incubated at room temperature for 15–20 min. Later, 200 μL of growth media was added and the plates were incubated at 12 C: Gills used L-15 supplemented with 5% FBS, 1% 100x AA solution, 1% 1 M HEPES, 1% 100x non-essential amino acids solution. Olfactory rosette explants had a similar composition as the media for the gills but without the non-essential amino acids.

### 2.4 Exposure to sodium hydrosulphide (NaHS) and mucus-interfering compounds

**FIGURE 1:**
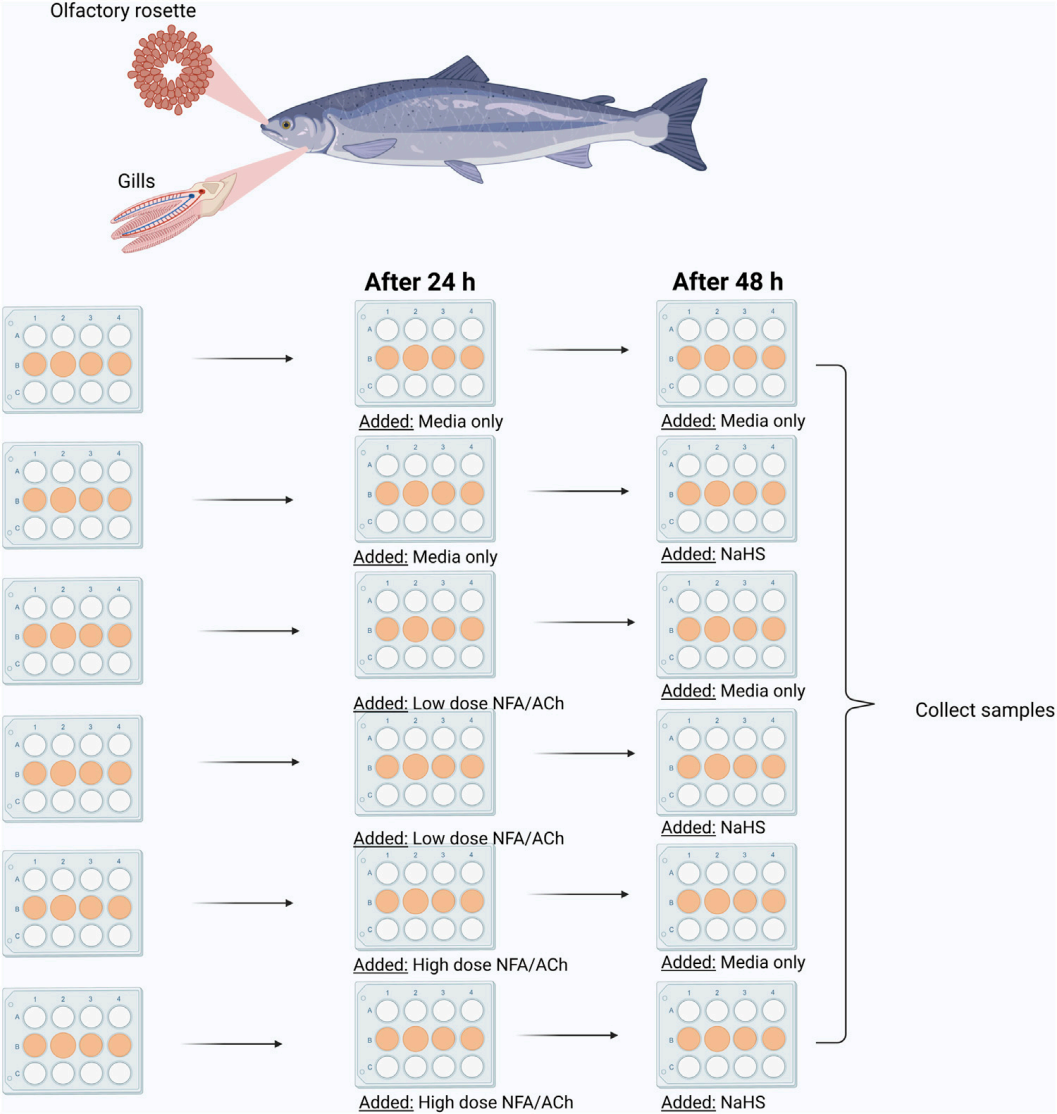
Graphic illustration of *in vitro* experimental set-up. Low NFA (50 μM); High NFA (500 μM); Low ACh (50 μM); High Ach (500 μM); NaHS (100 μM). Created with BioRender.com (Agreement number CU244LYF6H).

The study employed sodium hydrosulphide (NaHS) as the sulphide donor. The experimental set-up as described in Figure 1 aimed at analysing various effects: 1) the effects of sulphide donor alone on the mucosal explants; 2) the effects of acetylcholine chloride (ACh) or niflumic acid (NFA) on the mucosal explants; and 3) the effects of ACh or NFA pre-treatment on the responses of mucosal explants to the sulphide donor. ACh is a mucus stimulant while NFA is an inhibitor. H_2_S is considered a toxicant that targets the mucolytic function (Khattak et al., 2021); thus, the use of these compounds helped explain whether mucus interference would affect responses to the sulphide donor. These two organic compounds (ACh and NFA) have been previously used in pharmacological studies targeting mucus secretion in human and animal cell/tissue models (Zhou et al., 2002; Nakano et al., 2006; Módis et al., 2016).

Prior to NaHS exposure, a group of explants was incubated with either low (50 µM) or high (500 µM) NFA or ACh in growth media for 24 h at 12°C (Figure 1). Explants that would eventually receive NaHS only or served as the media control were handled similarly but by adding growth media alone. After the pre-treatment period, the media aspirated and replaced with new growth media for the media control group (control). Other explants were pre-treated with the mucus-interfering compounds (ACh/NFA only groups) or growth media containing 100 uM NaHS for groups that were pre-treated with the mucus-interfering compounds (ACh/NFA-NaHS group). Another separate group was not pre-treated (NaHS group). The plates were incubated for 24 h at 12°C, and samples for RNA isolation were collected thereafter. All samples were stored at −80°C prior to RNA isolation.

### 2.5 RNA isolation, quantification and cDNA synthesis

An Agencourt RNAdvance™ Tissue Total RNA Purification Kit (Beckman Coulter Inc., CA, United States) with the aid of Biomek 4000 automated workbench station (Beckman Coulter, Inc., CA, United States, Appendix IV) was used to isolate the total RNA from the samples. The isolated RNA was quantified using NanoDrop 8000 Spectrophotometer (ThermoFisher Scientific, United States). The quality of RNA samples was considered good if the nucleic acid-protein ratio was between 1.9–2.1. Consequently, the complementary DNA (cDNA) was synthesised from 500 ng template RNA using a High-Capacity cDNA Reverse Transcription Kit (Beckman Coulter, Inc., CA, United States) in a 20-μL reaction volume and thermocycling was carried out in a Veriti™ 96-Well Thermal Cycler 7 (Applied Biosystems, California, United States) with the following conditions: 10 min at 25°C, 30 min at 37°C, and 5 min at 95°C.

### 2.6 Real-time quantitative PCR and gene expression analysis

The quantification of gene expression was performed in a QuantStudio™ 5 Real-Time PCR system (Applied Biosystems) using Power SYBR Green PCR Master Mix (Applied Biosystems). Briefly, a 10 μL reaction mixture containing 4 μL 10X diluted cDNA, 5 μL of SYBR green, and 0.5 μL of each 10 µM forward and reverse primers of the target genes listed in Table 1. No RT control was included. All samples were run in duplicates with the following thermocycling conditions: 2 min of pre-incubation at 95°C; amplification with 40 cycles for 1 s at 95°C and 30 s at 60°C; and a melt curve stage of 15 s at 95°C, 1 min at 60°C, and 15 s at 95°C. The relative gene expression was calculated by the 2^–∆∆Ct^ method using the geometric mean of three housekeeping genes: *elongation factor 1-a* (*elf1a*), *β-actin* (*actb*), and *acidic ribosomal protein* (*arp*).

**TABLE 1:** Target and reference genes used for RT-qPCR.

Gene name	Abbreviation	Sequence (5′-3′)	Reference
*Acidic ribosomal protein*	*arp*	F: TCA​TCC​AAT​TGC​TGG​ATG​ACT​ATC	
		R: CTT​CCC​ACG​CAA​GGA​CAG​A	Sanden and Olsvik, (2009)
β *-actin*	*actb*	F: CCA​AAG​CCA​ACA​GGG​AGA​A	
		R: AGG​GAC​AAC​ACT​GCC​TGG​AT	Sanden and Olsvik, (2009)
*Elongation factor 1-* α	*elf1a*	F: GAA​TCG​GCT​ATG​CCT​GGT​GAC	
		R: GGA​TGA​TGA​CCT​GAG​CGG​TG	Garcia de la serrana and Johnston, (2013)
*Mucin 5ac-like*	*muc5ac*	F: GAC​CTG​CTC​TGT​GGA​AGG​AG	
		R: AGC​ACG​GTG​AAT​TCA​GTT​CC	Sveen et al. (2017)
*Mucin 5b-like*	*muc5b*	F: ATT​AAG​AGC​GAT​GTC​TTC​ACA​GC	
		R: AAG​CAC​ATG​AGT​CTC​TCA​CAC​AA	Sveen et al. (2017)
*Mucin 2-like*	*muc2*	F: GAG​TGG​GCT​CTC​AGA​TCC​AG	
		R: GAT​GAT​GCG​GAC​GGT​AGT​TT	Sveen et al. (2017)
*Sulphide:quinone oxidoreductase 1*	*sqor1*	F: GGA​TAG​GAA​GTA​TGA​TGG​CTA​CAC	
		R: GGT​CAA​TAG​GGA​ATG​TCT​CCA	This study
*Sulphide:quinone oxidoreductase 2*	*sqor2*	F: CCA​ACA​TCA​TGT​ACA​ACA​CGT​C	
		R: GCA​TCT​CAT​ACT​CAA​ACA​CTT​CAG	This study
*Sulfite oxidase*	*suox*	F: TGT​CTG​AGT​ATA​AGG​TGG​GTG​AG	
		R: GGT​GAT​GTA​GTT​GTC​GGA​GAG	This study

### 2.7 Data analysis

Significant differences in gene expression were analysed using SigmaStat Statistical Package (Systat software, London, United Kingdom). Assumptions for analysis of variance such as normality (Shapiro-Wilk) and variance (Brown-Forsythe) of the data sets were tested. If the tests failed, then they were log-transformed prior to analysis. Two-way ANOVA was used, and pairwise multiple comparisons were performed using the Holm-Sidak method. If the log-transformed data still failed the assumptions for analysis of variance, the Kruskal–Wallis ANOVA was used followed by a similar pairwise comparison procedure. All values are expressed as means with their corresponding standard deviation (SD). The level of significance was set to *p* < 0.05.

## 3 Results

### 3.1 Molecular responses of the gill explants to NaHS and niflumic acid

**FIGURE 2:**
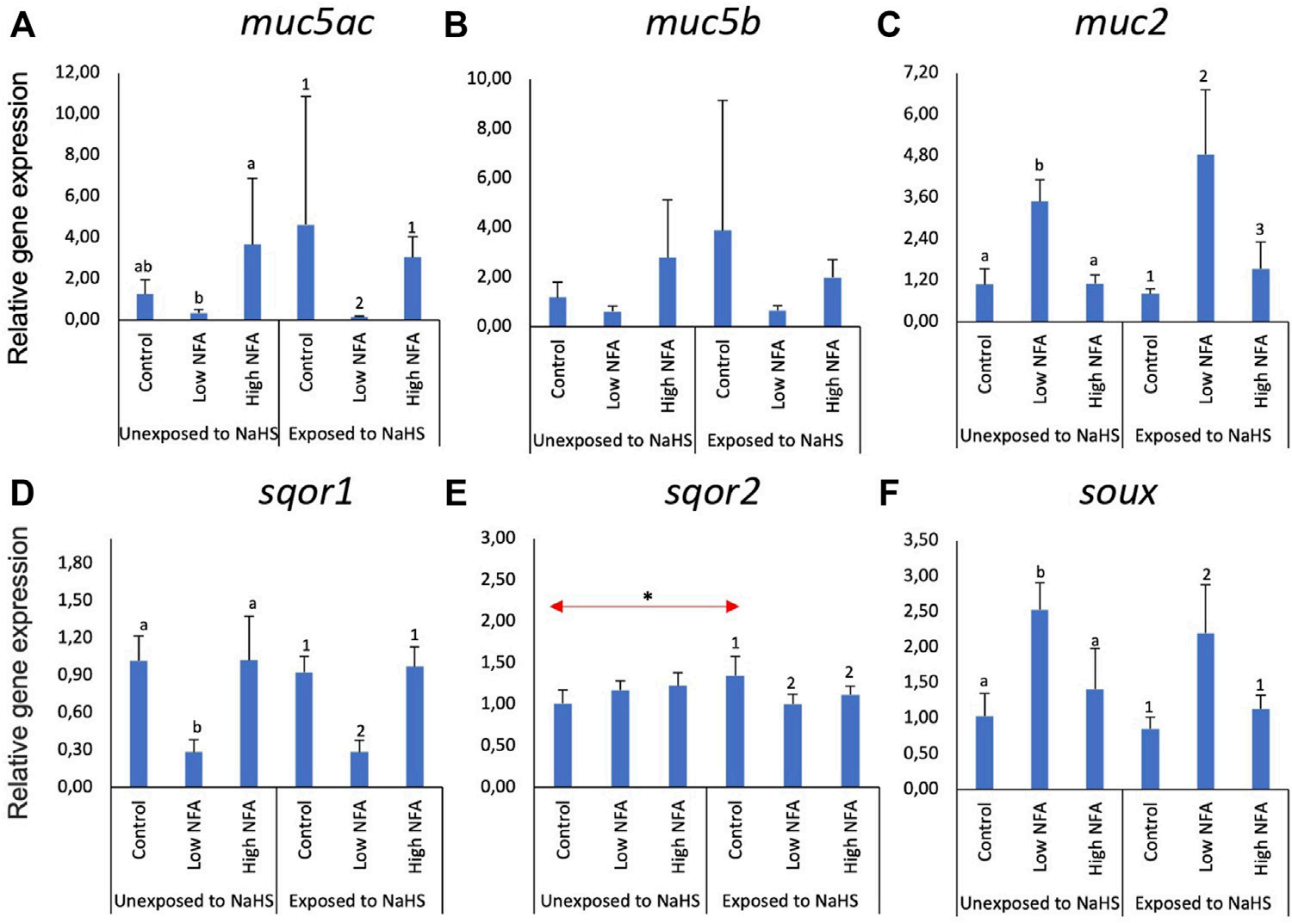
Relative gene expression of mucin genes **(A**–**C)** and sulphide-detoxification genes **(D**–**F)** in the gill explants treated with a mucus inhibitor Niflumic acid (NFA) with and without the NaHS exposure. The bars indicate mean and standard deviation of four individual fish (n = 4). Statistically significant differences (*p*-value<0.05) within unexposed to NaHS are denoted with letters, while numeric marks denote the differences within exposed to NaHS. Red arrows indicate significant differences in NaHS exposure (*p*-value<0.05).

Figure 2 shows the changes in the expression of three mucin (Figures 2A–C) and three sulphide detoxification (Figures 2D–F) genes in the gill explants following treatment with niflumic acid (NFA) and exposure to NaHS. The expression of *muc5ac* (Figure 2A) and *muc2* (Figure 2C) were significantly affected by NFA. The expression of *muc5ac* (Figure 2A) was significantly different between the two NFA-treated-NaHS-untreated groups in which the high dose group showed significantly elevated transcript levels compared to the low dose group. Non-etheless, neither group exhibited significant differences from the control group. These tendencies were not altered when the explants were eventually exposed to NaHS. The expression of *muc2* (Figure 2C) was significantly elevated in the groups that received a low dose of NFA *versus* the control and high-dose groups, regardless of whether they were exposed to NaHS or not. There were no significant changes on the expression of *muc5b* (Figure 2B) following NFA treatment and NaHS exposure.

The expression of sulphide detoxification genes, particularly *sqor1* (Figure 2D) and *soux* (Figure 2F), was affected by low dose NFA. This profile was not affected by exposure to NaHS. For *sqor1* (Figure 2D), the transcript level was significantly lower in the low-dose NFA group *versus* the control and high dose groups. An opposite tendency was identified in the expression of *suox* (Figure 2F), where the transcript level was significantly elevated in the low NFA group *versus* the control and high dose groups. The expression of *sqor2* (Figure 2E) was not significantly affected by NFA treatment in the groups unexposed to NaHS. However, there was a significant downregulation relative to control in the NFA-treated groups when the explants were exposed to NaHS (Figure 2E). Moreover, the expression of *sqor2* within the control groups showed a significant difference where the level in the group exposed to NaHS was significantly higher than those in the unexposed group.

### 3.2 Molecular responses of the olfactory rosette explants to NaHS and niflumic acid

**FIGURE 3:**
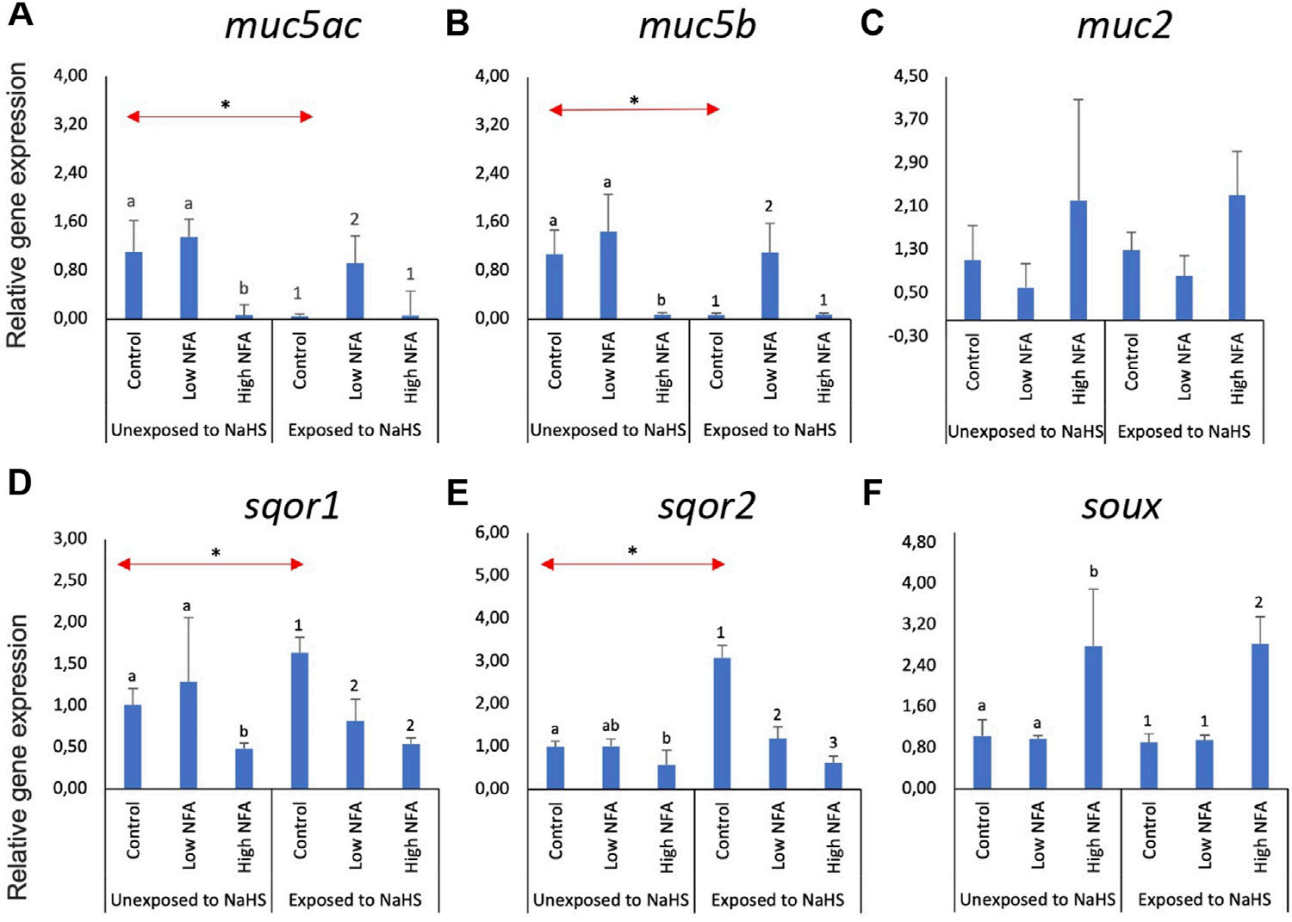
Relative gene expression of mucin genes **(A**–**C)** and sulphide-detoxification genes **(D**–**F)** in olfactory rosette explants treated with the mucus inhibitor Niflumic acid (NFA) with and without NaHS exposure. Statistically significant differences (*p*-value<0.05) within samples not exposed to NaHS are denoted by letters while numeric marks denote the differences within those exposed to NaHS. Red arrows indicate significant differences across NaHS exposure (*p*-value<0.05). Please note the differences in the *y*-axis.

The same set of genes was investigated in olfactory rosette explants (Figure 3). The expression of the two mucin genes, *muc5ac* (Figure 3A) and *muc5b* (Figure 3B), were significantly lower in the high NFA group *versus* the low and control groups when not subsequently exposed to NaHS. These tendencies were changed by exposure to NaHS where the transcript levels of *muc5ac* (Figure 3A) and *muc5b* (Figure 3B) were significantly elevated in the groups pre-treated with a low dose of NFA *versus* the control and high dose groups. Additionally, the expression of *muc5ac* (Figure 3A) and *muc5b* (Figure 3B) within the control groups were significantly downregulated when exposed to NaHS *versus* the NaHS-unexposed group. The expression of *muc2* (Figure 3C) did not show any significant changes following NFA treatment and NaHS exposure.

Without NaHS exposure, *sqor1* (Figure 3D) showed significantly lower expression in the high dose of NFA compared to the control and low dose groups. This trend was altered when the explants were exposed to NaHS: NFA-treated explants exposed to NaHS showed significantly lower *sqor1* expression *versus* controls regardless of the dose (Figure 3D). Within the groups unexposed to NaHS, the transcript levels of *sqor2* (Figure 3E) were significantly lower in the high-dose NFA-treated groups than the control. Subsequent treatment with NaHS resulted in the downregulation of *sqor2* expression (Figure 3E) in both NFA-treated groups *versus* controls. Within the control groups, treatment with NaHS resulted in the upregulation of *sqor1* (Figure 3D) and *sqor2* (Figure 3E) expression. The expression of *suox* (Figure 3F) was significantly upregulated in the high dose NFA groups compared to the low dose and control groups. This response profile was not influenced by the exposure to NaHS.

### 3.3 Molecular responses of the gill explants to NaHS and acetylcholine chloride

**FIGURE 4:**
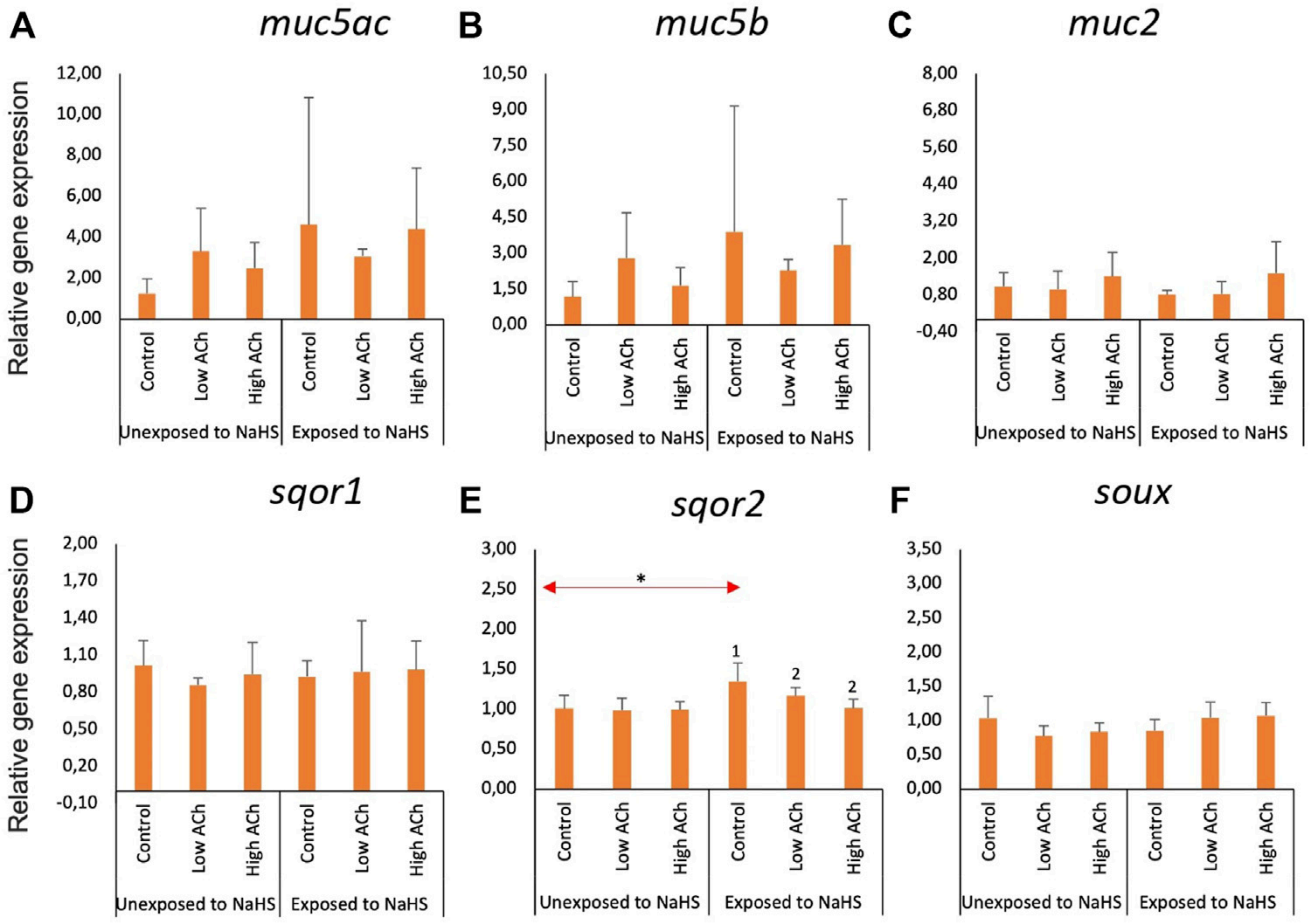
Relative gene expression of mucin genes **(A**–**C)** and sulphide-detoxification genes **(D**–**F)** in gill explants treated with acetylcholine chloride (Ach) with and without the NaHS exposure. The bars indicate mean and standard deviation of n = 4. Statistically significant differences (*p*-value<0.05) within samples unexposed to NaHS are denoted with letters while numeric marks denote the differences within exposed to NaHS. Red arrows indicate significant differences across NaHS exposure (*p*-value<0.05). Please note the differences in the *y*-axis.

The effects of NaHS and acetylcholine chloride (ACh) on the expression of mucin and sulphide detoxification genes in the gill explants are shown in Figure 4. Treatment with ACh did not significantly affect the expression of mucin genes (Figures 4A–C) or sulphide detoxification genes (Figures 4D–F) in the treatment groups except for *sqor2*. Following subsequent exposure to NaHS, the expression of *sqor2* (Figure 4E) was significantly downregulated in both groups pre-treated with ACh compared to the control. Moreover, the transcript level of *sqor2* (Figure 4E) within the control groups was upregulated following exposure to NaHS.

### 3.4 Molecular responses of the olfactory rosette explants to NaHS and acetylcholine chloride

**FIGURE 5:**
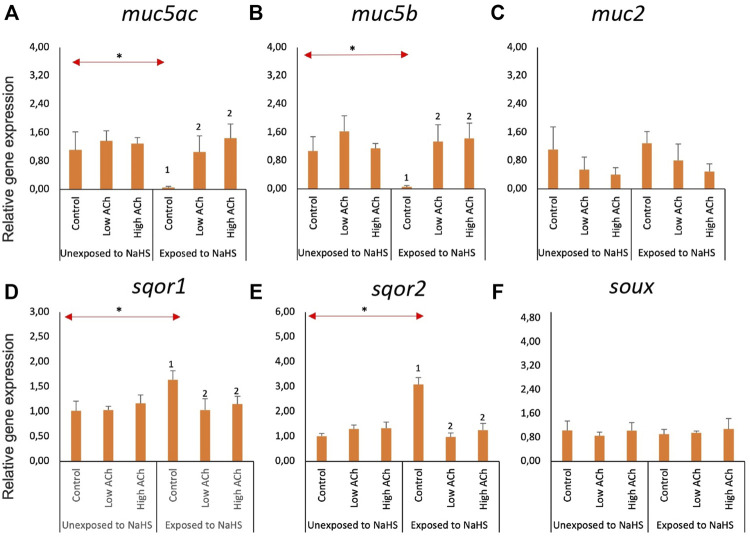
Relative gene expression of mucin genes **(A–C)** and sulphide-detoxification genes **(D–F)** in olfactory rosette explants treated with acetylcholine chloride (Ach) with and without the NaHS exposure. The bars indicate the mean and standard deviation of n = 4. Statistically significant differences (*p*-value<0.05) within samples unexposed to NaHS are denoted by letters while numeric marks denote the differences within samples exposed to NaHS. Red arrows indicate significant differences across NaHS exposure (*p*-value<0.05). Please note the differences in the *y*-axis.

Within the NaHS-unexposed group, the expression of the three mucin genes—*muc5ac* (Figure 5A), *muc5b* (Figure 5B), and *muc2* (Figure 5C) —was not significantly altered by ACh treatment. However, explants that were pre-treated with ACh, regardless of the dose, showed elevated transcription of *muc5ac* (Figure 5A) and *muc5b* (Figure 5B) upon exposure to NaHS. Moreover, the expression of these two genes within the control groups showed a significant downregulation upon exposure to NaHS. The expression of *muc2* (Figure 5C) was not significantly affected by ACh treatment and NaHS exposure.

The expression of *sqor1* (Figure 5D) and *sqor2* (Figure 5E) was not affected by ACh within the NaHS-unexposed group. However, exposure to NaHS resulted in a significantly lower expression of both *sqor1* and *sqor2* in groups pre-treated with ACh *versus* the control group. Within the control groups, the transcript level of *sqor1* and *sqor2* showed a significantly elevated level when exposed to NaHS *versus* the unexposed group. The expression of *suox* (Figure 5F) was not altered by ACh treatment and NaHS exposure.

## 4 Discussion

H_2_S is considered an irritant and has been shown to target the mucosa (Ammann, 1986; Richardson, 1995). Using organ explant models, we showed in this study that the two mucosal organs of Atlantic salmon, i.e., the gills and olfactory rosette, responded to a sulphide donor by modulating the expression of sulphide detoxification and mucin genes. These responses were influenced by prior exposure to mucus-interfering compounds, namely niflumic acid and acetylcholine chloride. To the best of our knowledge, this is the first report exploring such interactions in a fish model, thus offering new insights into the H_2_S-fish mucosa interactions.

Sulphide detoxification in fish mucosa is not well investigated particularly sulphides of exogenous origin. The mucosal organs of fish such as gills and olfactory organs have high sensitivity to external stressors because they are continuously interacting with the environment; thus, they are excellent models for understanding fish-H_2_S interactions. Sodium hydrosulphide, NaHS, is an inorganic salt, which is transformed into H_2_S upon exposure to water (Lee et al., 2011). Several studies have used NaHS as a sulphide donor in the establishment of H_2_S exposure model *in vitro* in a chicken hepatocellular carcinoma cell line (LMH) (Li et al., 2020); guinea pig, rat, and rabbit tissues (Teague et al., 2002); and human monocyte cell lines (Basic et al., 2017).

H_2_S is toxic at higher concentrations, and thus biological systems must have the mechanisms to detoxify this potential toxicant. One of these enzymes is called sulphide: quinone oxidoreductase (SQOR) (Wang, 2003; Bouillaud and Blachier, 2011). SQOR plays a primary role in the pathways governing the enzymatic detoxification of H_2_S (Hildebrandt and Grieshaber, 2008) and the previously described mitochondrial H_2_S oxidation pathway (Quinzii et al., 2017). These genes encode the membrane-bound SQOR enzyme that catalyses the initial two-electron oxidation of H_2_S into elemental sulphur *via* coenzyme Q as the electron acceptor. It simultaneously reduces a cysteine disulphide to produce a persulphide group—these are both non-toxic forms of sulphur (Theissen and Martin, 2008; Augustyn et al., 2017).

After 24 h of exposure to 100 μM NaHS, the expression of *sqor1* was significantly upregulated in olfactory rosette explants while the other isoform *sqor2* was significantly upregulated in both mucosal explant models. These two *sqor* genes may likely have organ-specific functions because they exhibited distinct regulation in both mucosal organ explants. Previous studies found that SQOR can use the sulphide for mitochondrial respiration and produce adenosine-triphosphate (ATP) at lower concentrations of NaHS (<10 μM). However, the H_2_S may inhibit cytochrome C oxidase (COX, complex IV) at concentrations over 10 μM and hence increase its toxicity in human cells (Di Meo et al., 2011; Módis et al., 2016). The upregulation of the *sqor* gene expression following exposure to the sulphide donor suggests a potential protective mechanism in the gills and olfactory organs of the Atlantic salmon against H_2_S toxicity. This further indicates that Atlantic salmon mucosa has innate characteristics for the detoxification H_2_S, thus, allowing them to thrive in an environment where stressors such as fluctuating levels of sulphide are encountered sporadically.

The mucus layer is a biophysical barrier and is an emblematic component of fish mucosa (Lazado and Caipang, 2014; Cabillon and Lazado, 2019). It protects the mucosa from external stressors such as toxicants, thus playing an important role in ensuring mucosal integrity. Mucins are the most abundant macromolecules in mucus (Gomez et al., 2013; Perez-Sanchez et al., 2013). There are several mucin-encoding genes in mammals (*muc5ac*, *muc5b*, *muc2*, *muc6* and *muc19*) and expressed in teleosts including in gilthead seabream (*Sparus aurata*), common carp (*Cyprinus carpio*), and Atlantic salmon (van der Marel et al., 2012; Perez-Sanchez et al., 2013; Lang et al., 2016). The two *muc5* genes, *muc5ac* and *muc5b*, play an essential role in airway defence in human and mouse, particularly in producing mucin glycoproteins that protect the respiratory surfaces from infections (Roy et al., 2014). Moreover, in humans, *muc5ac* is the dominant mucin gene expressed in goblet cells of the respiratory tract (Fahy, 2002). These molecules likely have a similar protective mechanism in nasal mucosa of salmon given that an earlier report from our group indicated their involvement in the nasal defence from oxidative toxicant (Osório et al., 2022). Here, out of the three mucin genes (*muc5ac*, *muc5b*, and *muc2*) examined *in vitro*, two of them (*muc5ac* and *muc5b*) showed a significant downregulation in olfactory rosette explants following exposure to NaHS. This implies that exogenous H_2_S may interfere with mucus production in the nasal mucosa of Atlantic salmon, which is probably a predisposing factor that may increase the mucosal immunotoxicity of H_2_S.

An gilthead seabream intestine, the downregulation of the mucin gene expression correlated with the reduction in the number of mucus-producing goblet cells (Perez-Sanchez et al., 2013). The downregulation of *muc5ac* and *muc5b* in olfactory organ explants shown here suggests potential impairment of mucosal function of the nasal mucosa to NaHS through interference of mucin expression. As a vital component of the mucosa, H_2_S-mediated alterations in mucosal functions through *muc5ac* and *muc5b* downregulation and may increase the susceptibility of Atlantic salmon to secondary stress and infection. A decrease in mucus secretion will also result in less non-specific humoral factors such as proteases, alkaline phosphatase, lysozymes, and other mucosal enzymes that are vital molecules in mucosal defence (Krasnov et al., 2012).

Does inhibition or stimulation of mucus production affect the responses of Atlantic salmon mucosa to H_2_S? We explored this question *via* pharmacological treatment of the explants by known drugs that have been shown to either inhibit or stimulate mucus production in mucosal surfaces. Niflumic acid (NFA) is a chloride-channel blocker that was previously demonstrated to inhibit mucus overproduction in human airway epithelial cells (Zhou et al., 2002; Nakano et al., 2006). Mucus production can be stimulated with TNF-α in human upper airway mucosal explant tissue; however, the addition of NFA can significantly reduce the mucus production in a dose-dependent way (Hauber et al., 2005). A higher NFA dose leads to lower mucus production. The main goal of NFA treatment was to understand whether inhibition of mucus production could impair the responses of mucosal organs to H_2_S. The low-dose NFA treatment significantly downregulated the expression of *muc5ac* and upregulated *muc2* in gill explants. This indicates that NFA may have dual functions in its interaction with mucins: This was likely dictated by mucosal organ type. This study showed that pre-treatment with NFA treatment followed by NaHS exposure did not affect the mucin gene expression for either mucosal tissue explants. Nevertheless, the results show that a low dose of NFA (50 μM) may essentially inhibit mucus secretion by suppressing the expression of *muc5ac* in olfactory rosette explants. A similar effect was found in another study where human airway epithelial cells exhibited low expression of *mu5ac* when exposed to NFA (Nakano et al., 2006). Additionally, a significantly reduced gene fold change of *muc5ac* was found in human upper airway mucosal explant tissue treated with 100 μM of NFA. Interference of the *muc2* gene expression may require higher NFA in the gill explants or NFA may not be a potent inhibitor of *muc2* but rather only *muc5* in Atlantic salmon. This is a working hypothesis that requires functional investigation in the future.

Another notable result was the upregulation of *muc5ac* and *muc5b* expression following treatment with low doses of NFA and NaHS in olfactory rosette explants. This suggests a protective mechanism of mucins in the olfactory mucosa from the toxic consequences of H_2_S despite the presence of a mucus inhibitor (NFA). NFA treatment differentially affected the expression of *sqor* genes in gill and olfactory rosette explants. Pre-treatment with NFA followed by exposure to NaHS significantly downregulated the expression of *sqor1* in olfactory rosette explant and *sqor2* in both organ explants. We speculate that the NFA treatment interfered with the detoxifying function of SQOR, which may be indicative of a decreased efficiency to resolve H_2_S-related toxicity in Atlantic salmon mucosa. This further illustrates that mucins are important components for mucosal defence against H_2_S.

Acetylcholine (ACh) is a known parasympathetic neurotransmitter that has been known to induce rapid secretion of mucus in the mammalian colonic mucosa particularly from goblet cells (Specian and Neutra, 1980). Here we used ACh to stimulate mucus secretion in mucosal organ explants to further illustrate the involvement of mucus in the H_2_S-mucosa interaction. The results revealed that the expression of *sqor* genes was downregulated when the explants were treated with ACh and consequently exposed to NaHS, thus implying a potential interference of ACh on H_2_S detoxification. Nevertheless, no increased response to NaHS was seen after ACh stimulation.

Stimulation of mucus secretion by ACh was not demonstrated in the gill explants. However, exposure to NaHS following low- or high-dose treatment of ACh considerably upregulated the expression level of *muc5ac* and *muc5b* in olfactory rosette explants. In a previous study, the treatment of 100 μM ACh significantly increased the gene expression of *muc5ac*, which triggers the formation of mucus in normal human bronchial epithelial cells after 48 h (Gundavarapu et al., 2012). The results suggest that ACh may improve the mucus functionality by modulating the mucin expression in the presence of NaHS, thus providing a potential protection from H_2_S-related toxicity in the nasal mucosa.

This study demonstrated that the sulphide donor modulated the expression of genes involved in sulphide detoxification and mucins in the mucosa of Atlantic salmon. The gene expression data suggest that Atlantic salmon mucosa have a H_2_S-detoxifying ability based on the transcriptional changes of key genes with crucial role in sulphide detoxification following exposure to NaHS. Mucins play an important role in protecting the mucosa from H_2_S. This was demonstrated here by how mucus-interfering compounds modulated the expression of key marker genes following sulphide treatment. An *in vivo* exposure study will further elucidate how mucosal organs of salmon respond and adapt to either acute or chronic exposure to H_2_S. This study contributes to our understanding of how the enigmatic H_2_S modulates mucosal functions of Atlantic salmon and how the genes may be explored as potential marker genes for mucosal responses to H_2_S.

## Data Availability

The raw data supporting the conclusions of this article will be made available by the authors, without undue reservation.
